# Exploring arterial tissue microstructural organization using non-Gaussian diffusion magnetic resonance schemes

**DOI:** 10.1038/s41598-021-01476-z

**Published:** 2021-11-15

**Authors:** Syed Salman Shahid, Robert D. Johnston, Celine Smekens, Christian Kerskens, Robert Gaul, Brooke Tornifoglio, Alan J. Stone, Caitríona Lally

**Affiliations:** 1 Department of Radiology and Imaging Sciences, Indiana University School of Medicine, Indianapolis, IN USA; 2 Indiana Alzheimer Disease Research Center, Indiana University School of Medicine, Indianapolis, IN USA; 3 Trinity Centre for Biomedical Engineering, Trinity Biomedical Sciences Institute, Trinity College Dublin, Dublin, Ireland; 4 Department of Mechanical, Manufacturing and Biomedical Engineering, School of Engineering, Trinity College Dublin, Dublin, Ireland; 5 Trinity College Institute of Neuroscience, Trinity College Dublin, Dublin, Ireland; 6 Advanced Materials and Bioengineering Research Centre (AMBER), Royal College of Surgeons in Ireland and Trinity College Dublin, Dublin, Ireland

**Keywords:** Biomarkers, Engineering, Biomedical engineering, Medical research, Preclinical research

## Abstract

The purpose of this study was to characterize the alterations in microstructural organization of arterial tissue using higher-order diffusion magnetic resonance schemes. Three porcine carotid artery models namely; native, collagenase treated and decellularized, were used to estimate the contribution of collagen and smooth muscle cells (SMC) on diffusion signal attenuation using gaussian and non-gaussian schemes. The samples were imaged in a 7 T preclinical scanner. High spatial and angular resolution diffusion weighted images (DWIs) were acquired using two multi-shell (max b-value = 3000 s/mm^2^) acquisition protocols. The processed DWIs were fitted using monoexponential, stretched-exponential, kurtosis and bi-exponential schemes. Directionally variant and invariant microstructural parametric maps of the three artery models were obtained from the diffusion schemes. The parametric maps were used to assess the sensitivity of each diffusion scheme to collagen and SMC composition in arterial microstructural environment. The inter-model comparison showed significant differences across the considered models. The bi-exponential scheme based slow diffusion compartment (Ds) was highest in the absence of collagen, compared to native and decellularized microenvironments. In intra-model comparison, kurtosis along the radial direction was the highest. Overall, the results of this study demonstrate the efficacy of higher order dMRI schemes in mapping constituent specific alterations in arterial microstructure.

## Introduction

Alteration in arterial microstructure is a key manifestation of cardiovascular diseases including atherosclerosis and aneurysms. As disruption in tissue microstructure can alter diffusion of water molecules in a tissue microenvironment^1–4^, diffusion magnetic resonance imaging (dMRI) can be used to non-invasively probe tissue microstructure and quantify pathology associated microstructural alterations. The dMRI derived quantitative parameters can detect the presence of multiple microstructural constituents based on their structural characteristics to restrict and or hinder water molecular diffusion^5,6^. By identifying alterations in arterial tissue microstructure, dMRI may provide a mechanism for the early diagnosis of cardiovascular disease.

In order to realize this potential, it is important to understand the influence of the key microstructural components of arterial tissue on diffusion attenuated signal. Structurally, arterial vessels are composed of three layers namely: intima, media and adventitia. Each layer is made up of a combination of collagen, elastin, glycosaminoglycans (GAGs) and vascular cells. The most abundant cell type in arteries is the collagen and elastin producing smooth muscle cells (SMC)^7^. The most significant load bearing component within an arterial tissue is collagen which is woven in a helical pattern throughout the vessel^7^. Collagen is critical to maintaining vessel integrity^8–12^ and collagen remodeling, or lack thereof, is considered to play a significant role in vascular disease, including the onset and progression of atherosclerosis and subsequent plaque stability^13^, restenosis^14^ and the development of aneurysms^15^. Interestingly, SMC and collagen fibers align themselves with each other and in the media of healthy arteries, SMCs are considered to be enveloped by collagen^16^. Given that collagen production from vascular cells is critical for stability, the presence of SMCs is an indication of vessel’s ability to remodel and maintain such stability^17^.

Since the first feasibility study on arterial tissue conducted by our group in 2010^18^, there has been a steady growth in the applications of dMRI to in vitro arterial tissue^19^, tissue engineered vascular tissues^20^ and even some recent preliminary in vivo studies in a healthy population^21,22^. These studies have identified dMRI as a means to measure the collagen architecture of arteries. From healthy porcine carotid arteries^18,23^ to human endarterectomy plaques^19^, collagen structure as well as changes to the tissue structure due to alterations in the integrity of the tissue, i.e., frozen, cut-open, flattened^24,25^, have been examined. By comparing with histology and second harmonic generation (SHG), these above mentioned studies have suggested that collagen structure can be mapped by estimating the diffusion profile of water molecules in arterial tissue^23^. Additionally, some of the studies reported that changes in the arterial microstructure can be estimated by the directionally variant apparent diffusion coefficient (ADC)^26–30^ or by mapping the self-diffusion profile using a mono-exponential signal representation scheme^25,31^. To date, only one study by our lab has tried to estimate the contribution of individual microstructural constituents under the assumption of gaussian diffusion paradigm^31^. Despite the oversimplification, the study showed that the diffusion magnetic resonance (dMR) signal in arterial tissues is heavily attenuated by SMC, not the collagen.

Random motion of water molecules in arterial tissue microstructure is hindered and restricted due to the interaction of these molecules with tissue components such as macromolecules, cells, elastin and collagen fibers. These interactions cause the diffusion profile in a microenvironment to deviate from Gaussian distribution. To correctly characterize non-Gaussian behavior and to map the contributions of these components to dMR signal attenuation it would be prudent to assess the contribution of individual components on diffusion signal at high spatial and angular resolutions. To the best of our knowledge, there are no studies to date that have addressed the non-Gaussian behavior of water diffusion in arterial tissue. Thus, the aim of this study was to better characterize the microstructural organization of arterial tissue under various induced structural offsets. To achieve this task, porcine carotid arteries (PCaAs) were obtained and enzymatically treated in one of the following ways prior to imaging: (1) native (untreated intact ring samples), (2) collagenase treated ring samples to selectively remove collagen, and (3) decellularized PCaA ring samples by selectively removing SMCs. Using high spatial and angular resolution multi-shell dMRI, the study aimed to elucidate the differences between Gaussian and non-Gaussian based microstructural profiles of native and treated PCaAs. We hypothesize that the non-Gaussian estimation schemes are more sensitive to alterations in tissue microstructure than Gaussian estimation and may serve as a sensitive biomarker for the early detection of CVD.

## Materials and methods

### Tissue preparation

Fresh porcine common carotid arteries were excised from 6-month-old Large White pigs weighing approximately 80 kg at the time of slaughter. Carotid arteries were transported on ice and frozen to − 80 °C at a controlled rate of − 1 °C/min in the presence of a cryoprotectant to maintain mechanical and structural properties^32^. Native tissue samples were preserved for a period of 2–3 weeks prior to imaging. To investigate the effect of collagen, collagenase was used to selectively remove collagen without compromising the integrity of the tissue or its cellular contents. Three PCaA samples were incubated in Type I purified bacterial collagenase (400 U/ml; Worthington Biochemical, USA) in PBS supplemented with calcium and magnesium (D8662; Sigma-Aldrich, Ireland) for 42 h to completely and selectively remove the collagen^33^ (confirmed by histology). Three cryopreserved PCaAs were taken out from − 80 °C storage and thawed fast to avoid any cellular damage by the dimethyl sulfoxide (DMSO), a component in the cryopreservation media. An in-house optimized decellularization protocol based on a short-term chemical decellularization method described in Campbell et al.^34^ was adopted for the removal of cells from PCaAs. First, the thawed porcine tissue was perfused with a 0.1 M sodium hydroxide (NaOH) solution for 15 h to disrupt the cells. Secondly, a 0.9% sodium chloride (NaCl) solution was used to wash the tissue for 30 h to remove NaOH solution and cellular debris. Within this period of time, the NaCl solution was replaced twice. To improve the infiltration of the solutions along the length of the artery, a pulsatile perfusion system was employed in which the three 40 mm long PCaAs were mounted horizontally in a flow chamber with nylon cannulas inserted into the arterial lumen at either end. An overhead fluid reservoir maintained a pressure of 100 mmHg in the flow chambers for the duration of the decellularization process. A peristaltic pump (323, Watson Marlow, UK) circulated the solutions at 80 rpm. Prior to filling the flow chambers with fluid, the vessels were assessed for leaks. Once the NaCl rinsing process was completed, the vessels were taken out of the flow chambers and immersed in a DNAse digestion solution (3Kunits/ml Worthingtons DNAse I in 1 × Promega reaction buffer, supplemented with Primocin) to remove all remaining cellular debris. The fully submerged tissue was incubated for 19 h at 37 °C and subsequently transferred into cryopreservation medium for − 80 °C storage until imaging.

### Histology

**Figure 1:**
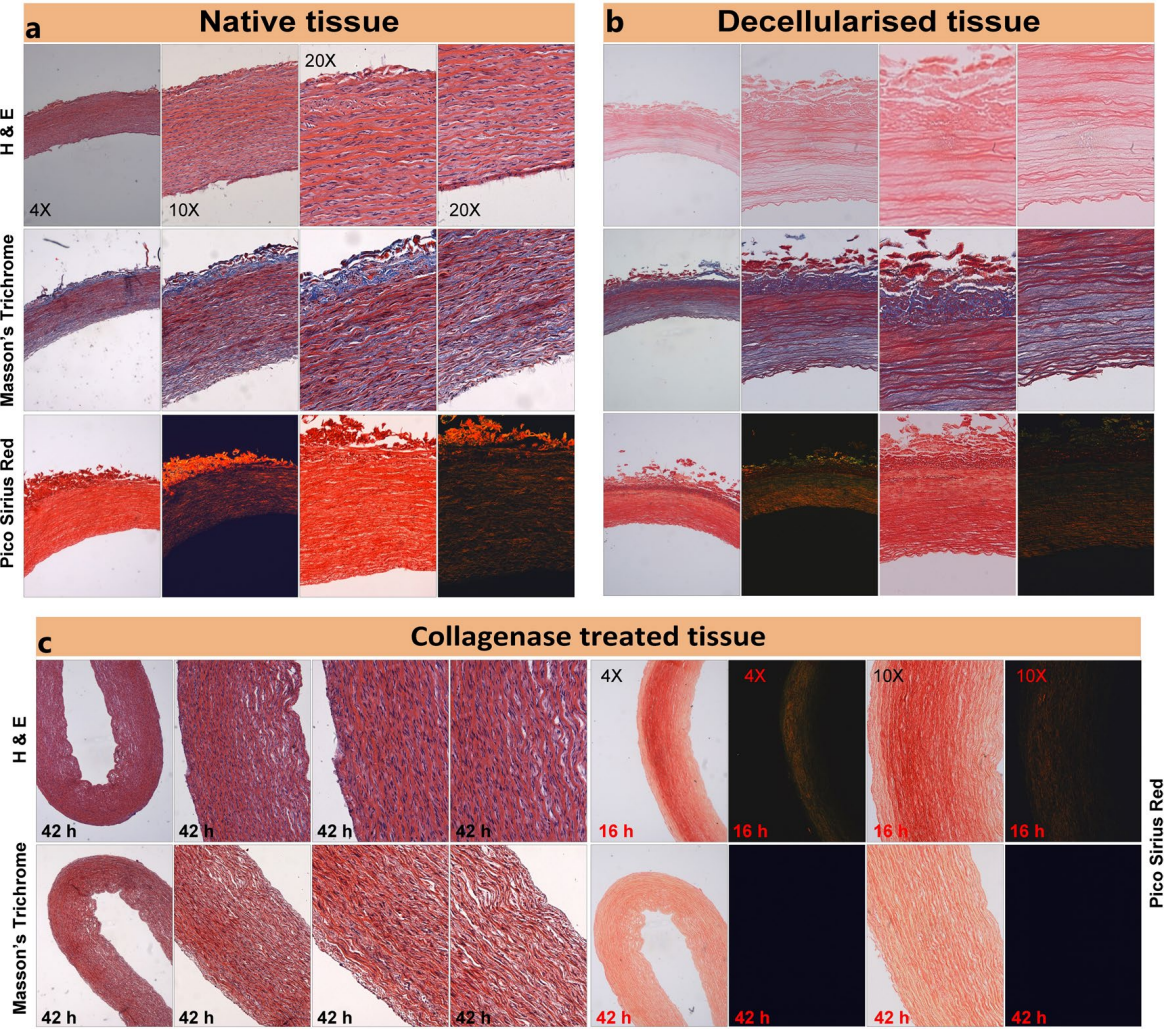
Histological ring sections of porcine common carotid artery. (**a**) Native tissues, (**b**) decellularized tissues and (**c**) collagenase treated tissues were stained with hematoxylin and eosin (H&E) to visualize the cell density. Picrosirius red stain (PSR) was used to visually observe the collagen content under polarized light and Masson’s Trichrome stain was used to further validate the observations. (Histological images are from different samples and are representative images).

Native, collagenase treated and decellularized PCaA samples were separately fixed in 10% formalin at 4 °C for 12 h. The samples were dehydrated and then exposed to paraffin before the final embedding of the samples in paraffin blocks. 5 µm axial cross sections were sliced from each sample and floated on distilled water at 37 °C before being mounted on glass slides. Once mounted, these slides were then left to dry overnight. Native, collagenase and decellularized samples were treated with Picrosirius red stain to visually observe the collagen content, hematoxylin and eosin (H&E) stain to visualize the cell density and Masson’s Trichrome stain to visualize collagen, in order to further validate the effects of each treatment. These slides were imaged using polarized light microscopy (Leica, Wetzlar, Germany) at a range of magnifications (Fig. 1).

### MR imaging

The diffusion weighted images (DWIs) and anatomical images were acquired in a horizontal bore 7 T Biospec micro-MRI system (Bruker, Etlingen Germany) equipped with shielded gradients (maximum gradient strength = 770 mT/m, rise time = 115 µs) and ^1^H mouse cryogenic surface coil (cryoProbe, Bruker Biospin). All MRI protocols were acquired in a single session and the same sample was used for DWI and anatomical acquisitions. Two multi-shell acquisitions were used for DWIs, namely: high multi b-value (HMb) and high angular resolution (HAR) protocols. The common acquisition parameters were as follows: 2D spin echo-based diffusion sequence with a multi-segmented EPI readout and unipolar diffusion sensitizing pulse field gradients placed symmetrically around the 180° RF pulse. Field of view (FOV) = 13 × 13 mm^2^, matrix size = 128 × 128; slice thickness 0.3 mm; voxel size = 101.5 × 101.5 × 300 µm^3^; no inter-slice spacing; number of slices = 12; average = 1 and no fat suppression. HMb protocol specific acquisition parameters were as follows: echo time (TE)/repetition time (TR) = 29.09/3000 ms; diffusion duration (δ) = 3.55 ms; diffusion time (Δ) = 8.52 ms; 12 b values ranging from 0 to 3000 s/mm^2^ (100, 200, 300, 600, 900, 1200, 1500, 1800, 2100, 2400, 2700, 3000) s/mm^2^, 121 b0 (non-DW images) and 12 diffusion encoding directions per shell. HAR protocol specific acquisition parameters were as follows: TE/TR = 27.50/3000 ms; δ/Δ = 3.55/8.52 ms. Three shells were used in HAR protocol (b = 800, 1600 and 2200 s/mm^2^), there were 96 diffusion encoding directions per shell and gradient polarity reversal was used to suppress gradient cross-terms^35,36^. In total there were 20 b0 images per shell and a total of 192 diffusion gradient directions per shell. To minimize bias due to heat transfer from the gradient system, each DWI acquisition was pseudo-randomized such that consecutive DWI scans were acquired with widely different b-values, and DWI and b0 scans were interleaved^37^. Nyquist ghosting, phase fluctuations and frequency drifts were compensated by including additional EPI scan parameters such as double sampling, automatic trajectory adjustment and regridding based on EPI trajectory. The total scan time for HMb acquisition was 3 h. The total scan time for HAR acquisition was 7 h.

The values of δ and Δ were selected based on our previous work^25^ and in-house quantitative T2 analysis. Using 2D multi-echo spin echo sequence, the T2 spectra from tunica media was obtained by voxel-wise fitting of multi-exponential T2 curve using non-negative least squares with a regularization factor (data and procedure not shown). The qT2 of native tissue was estimated to be in the range of 40 ms, for collagenase treated samples, the T2 was close to 38 ms and for decellularized tissues, qT2 was estimated to be close to 35 ms. To accommodate TE (≈ 27–29 ms), close to the T2 of the tissue and to improve the SNR of the acquisition, the above stated values of δ and Δ were selected.

For each sample, high resolution T1-W (Bruker T1FLASH) images were also acquired using the following parameters: TE/TR = 7.0/409.8 ms, flip angle = 30°, number of slices = 12, slice thickness = 0.3 mm, FOV = 13 × 13 mm^2^; matrix size = 512 × 512; voxel size = 25.39 × 25.39 × 300 µm^3^; number of averages = 20 and total acquisition time of 46 min.

### dMRI data processing and modelling

The magnitude DWI data was processed to reduce the effect of noise. The noise estimation was carried out by exploiting the inherent redundancy in dMRI data^38,39^. At low signal to noise ratio (SNR < 3), the positive signal bias was removed by using the denoised signal as a proxy for the Rician expectation value^40^. The processed datasets (denoised and Rician bias corrected) were then corrected for Gibbs ringing artefact^41^, for eddy current induced distortions^42^ and for B1 field inhomogeneity^43^. 

For each dataset, the region of interest (ROI) was manually traced using the sample’s averaged b0 image. In each case, the ROI was only traced in the media of the samples due to the fact that media is the most significant load bearing region of a vessel and it is also the region most associated with remodeling^10^. To account for intra-sample heterogeneity in the media microstructure, 4 slices per sample were selected for model fitting and further analysis. The processed DWIs (native, collagenase treated and decellularized) were fit in the defined ROIs using the following four statistical models of diffusion:

1 $$\ln \frac{S(b)}{{S(0)}} \approx - bD_{app}$$

where Eq. (1) represents mono-exponential model^44^; S(b) is the diffusion attenuated signal at a particular b-value and S(0) is the signal at b = 0 s/mm^2^. D_app_ is the apparent diffusivity.

2 $$\frac{S(b)}{{S(0)}} = e^{{ - b^{\alpha } D_{st} }}$$

where Eq. (2) represents stretched-exponential model^45^; α is a dimensionless stretching parameter or a heterogeneity index (0 < α ≤ 1) and D_st_ is the stretched-regulated diffusivity. α characterizes the deviation of the diffusion-weighted signal from mono-exponential behavior. α close to 0 describes non-mono-exponential behavior due to underlying microstructural complexity (heterogeneity), or in other words a higher degree of kurtosis, and α close to 1 indicates a higher degree of homogeneity in the diffusion profile.

3 $$\ln \frac{S(b)}{{S(0)}} \approx - bD_{app} + \frac{1}{6}b^{2} D_{app}^{2} K_{app}$$

where Eq. (3) represents diffusion kurtosis model^46^; D_app_ is the apparent diffusivity and K_app_ is apparent diffusion kurtosis.

4 $$\frac{S(b)}{{S(0)}} = v_{f} e^{{ - D_{f} b}} + v_{s} e^{{ - D_{s} b}}$$

where Eq. (4) represents bi-exponential model^47^; v_f_ and v_s_ are the volume fractions corresponding to fast D_f_ and slow D_s_ diffusion compartments, respectively and sum of v_f_ and v_s_ is unity. Under the assumption that D_f_ ≥ D_s_, the mean diffusivity (D_biexp_) and kurtosis (K_biexp_) can be calculated as: D_biexp_ = v_f_D_f_ + v_s_D_s_ and K_biexp_ = 3v_f_v_s_(D_f _− D_s_)^2^/D^2^_biexp_^46^.

**Figure 2:**
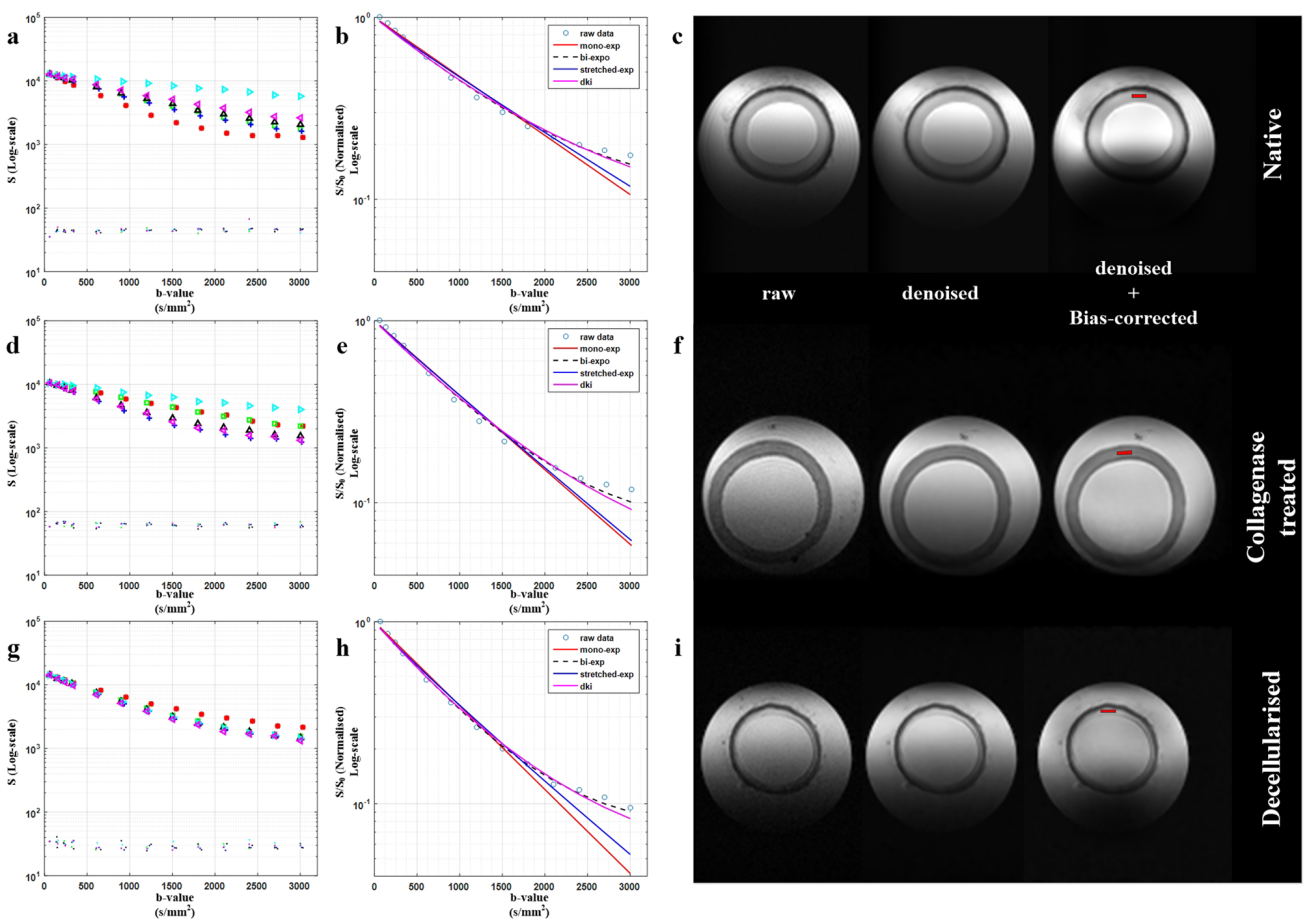
Diffusion-weighted signal decay curves as a function of b-value from the six non-collinear diffusion-encoding directions. Profile of noise corrected diffusion-weighted signal decay curves across six diffusion-encoded directions (HMb protocol) for (**a**) native tissue, (**d**) collagenase treated tissue and (**g**) decellularized tissue samples. In (**a**, **d**, **g**), the calculated signal noise floor is also shown. (**b**, **e**, **f**) The comparison of various signal representation based fitting schemes on the actual noise and bias corrected diffusion-weighted signal (for illustration purpose, the DW-signals from the sixth diffusion-encoded direction were used in **b**, **e** and **h**). (**c**, **f**, **i**) The b0 images of native, collagenase treated and decellularized tissues samples, respectively. The noise floor was calculated using the standard deviation of the background signal from a region (16 × 16 × 16 voxels) within the “Reference Power band”.

Additional details pertaining to dMRI modelling is provided in the supplementary section. For HMb protocol, non-linear least squares fitting, using the Trust-region reflective algorithm with positive constraints, was used (implemented in Matlab) for all model fitting. The initial estimates for mono exponential and DKI models were obtained using the linear least square fitting scheme. The initial estimates for the stretched-exponential model were derived from the mono-exponential fit (α = 1) with the lower and upper bound of α in the range of 0.1–1.0. In the bi-exponential model, the coefficients for fast and slow diffusion were estimated using mono-exponential fits for b ≤ 900 s/mm^2^ (D_f_) and b ≥ 1800s/mm^2^ (D_s_), respectively. Each model was initially tested and validated on simulated data. Supplementary Fig. 1 shows the efficacy of each model’s fit to simulated diffusion decay data. Figure 2 and supplementary Fig. 2 demonstrate the fitting behavior of each model on real denoised diffusion attenuated signals.

For each model under HMb protocol, the assessments were carried out using the directional analysis and for each non-gaussian model it was assumed that each model shares the same frame of reference as that of the mono-exponential model^48–50^. Using HMb protocol, directionally invariant imaging indices such as fractional anisotropy (FA), mean diffusivity (MD) and directionally variant diffusivity parameters such diffusion along primary (*v*_*1*_), secondary (*v*_*2*_) and tertiary (*v*_*3*_) eigenvectors were derived from the DTI (mono-exponential) model (Eq. 1). From Eq. (2), *D*_*st*_ and α were calculated along *v*_*1*_, *v*_*2*_ and *v*_*3*_. From Eq. (3), diffusivity and kurtosis along *v*_*1*_, *v*_*2*_ and *v*_*3*_ were calculated and from Eq. (4), *D*_*f*_ and *D*_*s*_ along *v*_*1*_, *v*_*2*_ and *v*_*3*_ were calculated.

Since DKI is sensitive to phase fluctuations due to scanner associated mechanical vibrations and higher angular frequencies associated with the omitted higher terms of the Taylor series (see supplementary dMRI model fit Eq. 4) can also impact DKI assessment, high angular resolution (HAR) data (b = 0, 800, 1600 and 2200 s/mm^2^) were also used for DTI and DKI assessment. HAR based assessments also served as a baseline to validate the DTI and DKI based results from HMb protocol (low angular resolution). For HAR, the DTI assessment was done with b ≤ 800 mm/s^2^, whereas for DKI the entire HAR range was used. For HAR based DWIs, ExploreDTI was used^51^ and microstructural imaging indices such as FA, MD and three geometric measures (Westin shapes) were generated from DTI^52^. Westin shapes, namely Linear (*C*_*L*_), Planar (*C*_*P*_) and Spherical (*C*_*P*_) use the symmetric properties of an ellipsoid to represent the geometric shape of a second rank symmetric tensor^52^. DKI was used to extract kurtosis anisotropy (KA), mean kurtosis (MK), axial kurtosis (AK), radial kurtosis (RK) and tortuosity (TORT). KA estimates the variability in the kurtosis and is derived from the standard deviation of the kurtosis. KA has a range from 0 to ∞ and it captures diffusional anisotropy with the additional dimensionality that is provided by the kurtosis model. MK measures the average diffusion kurtosis along all directions, AK measures diffusion kurtosis along the principal diffusion direction and RK measures kurtosis transverse to the principal diffusion direction. Higher values in MK, AK and RK represent increased restriction to molecular diffusion (non-gaussian) along those dimensions.

To quantify the influence of collagen and SMC on diffusion profile, the gaussian and non-gaussian based indices from native, collagenase treated and decellularized samples were compared using one-way analysis of variance (ANOVA) in GraphPad Prism (Version 6). Post-hoc tests were performed to further explore the sensitivity of diffusion derived indices to microstructural alterations in group-wise analysis. Differences were considered significant when *p* < 0.05. Results are listed as mean ± standard deviation.

## Results

### Histology

It can be observed from Picrosirius red (PSR) staining of the native and collagenase treated samples that the collagen content gradually reduced after exposure to the collagenase solution over a time-period of 42 h (Fig. 1a, c—Picrosirius red). Compared to native tissue the light microscopy images show a decrease in red stain for collagen after the exposure. The polarized light microscopy (PLM) images also show a decrease in collagen content with increased exposure to collagenase solution (Fig. 1c, Picrosirius red). The H&E staining in the native samples show that the cells are dense and aligned with the collagen structure (Fig. 1a, H&E). However, following decellularization, cells are no longer present throughout the tissue (Fig. 1b, H&E). It can also be observed that after decellularization, the collagen structure remains intact (Fig. 1c, H&E). These observations were validated by Masson’s Trichrome stain. For the native samples, considerable collagen content (blue) and cell density (black) is evident (Fig. 1a, Masson’s Trichrome). Lack of cells and nuclear material (black) is evident in decellularized samples (Fig. 1b—Masson’s Trichrome). Visual comparison between native and collagenase treated samples show reduction in collagen with extended exposure to collagenase and lack of collagen after 42 h (Fig. 1a, c—Masson’s Trichrome).

### Gaussian and non-gaussian diffusion profiles

**Figure 3:**
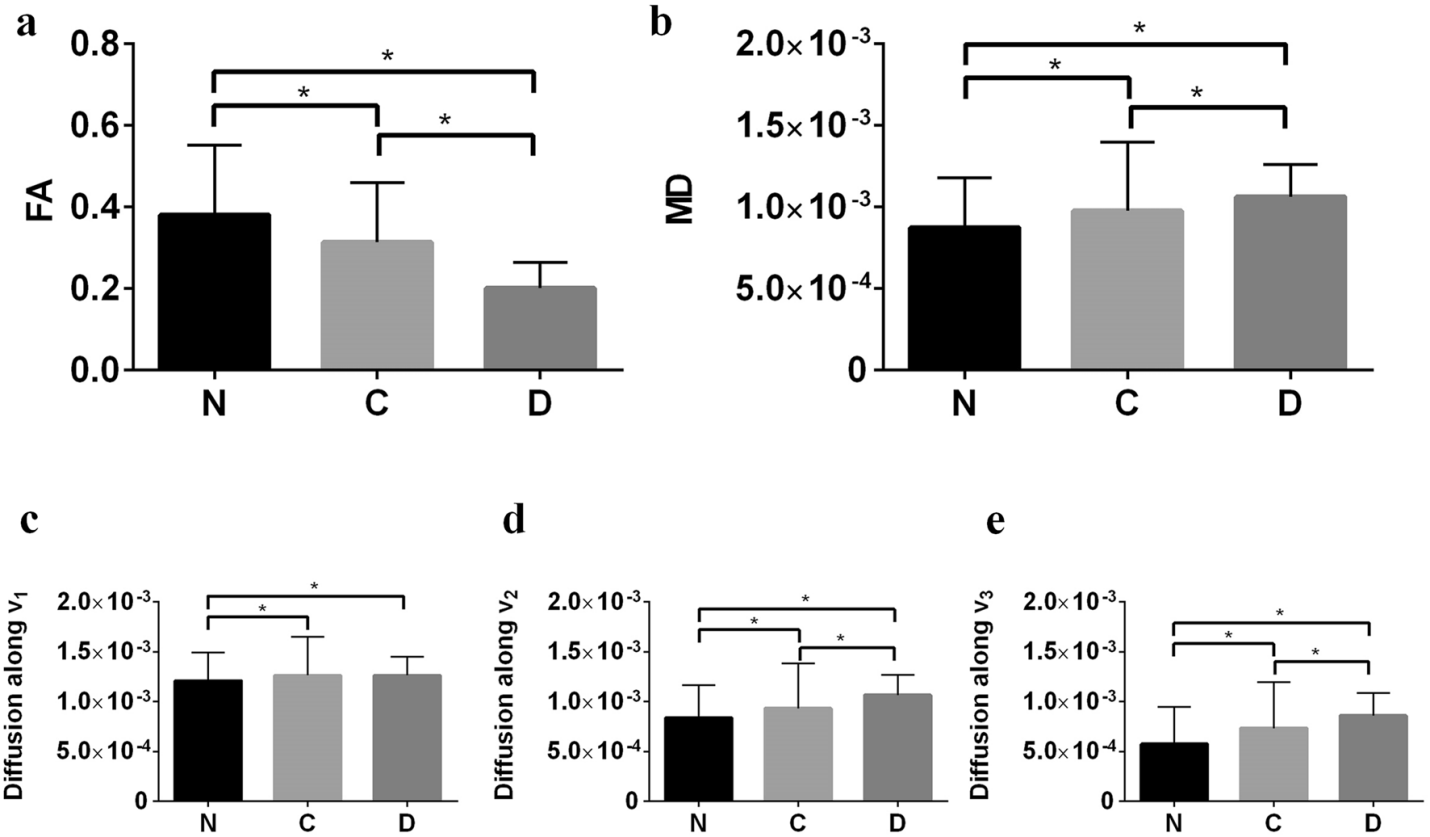
Quantification of tissue heterogeneity using DTI based indices. The derived indices are displayed as columns (mean) for each ROI. Three native tissue (N) samples, three collagenase treated tissue (C) samples and three decellularized tissue (D) samples were used for comparison. (n = 12, one ROI per slice, 4 slices per sample). (**a**) Fractional anisotropy (FA), (**b**) mean diffusivity (MD), (**c**) diffusion along V_1_, (**d**) diffusion along V_2_ and (**e**) diffusion along V_3_. Values represent mean ± SD. *p < 0.05. HMb protocol was used to calculate the DTI derived indices.

**Figure 4:**
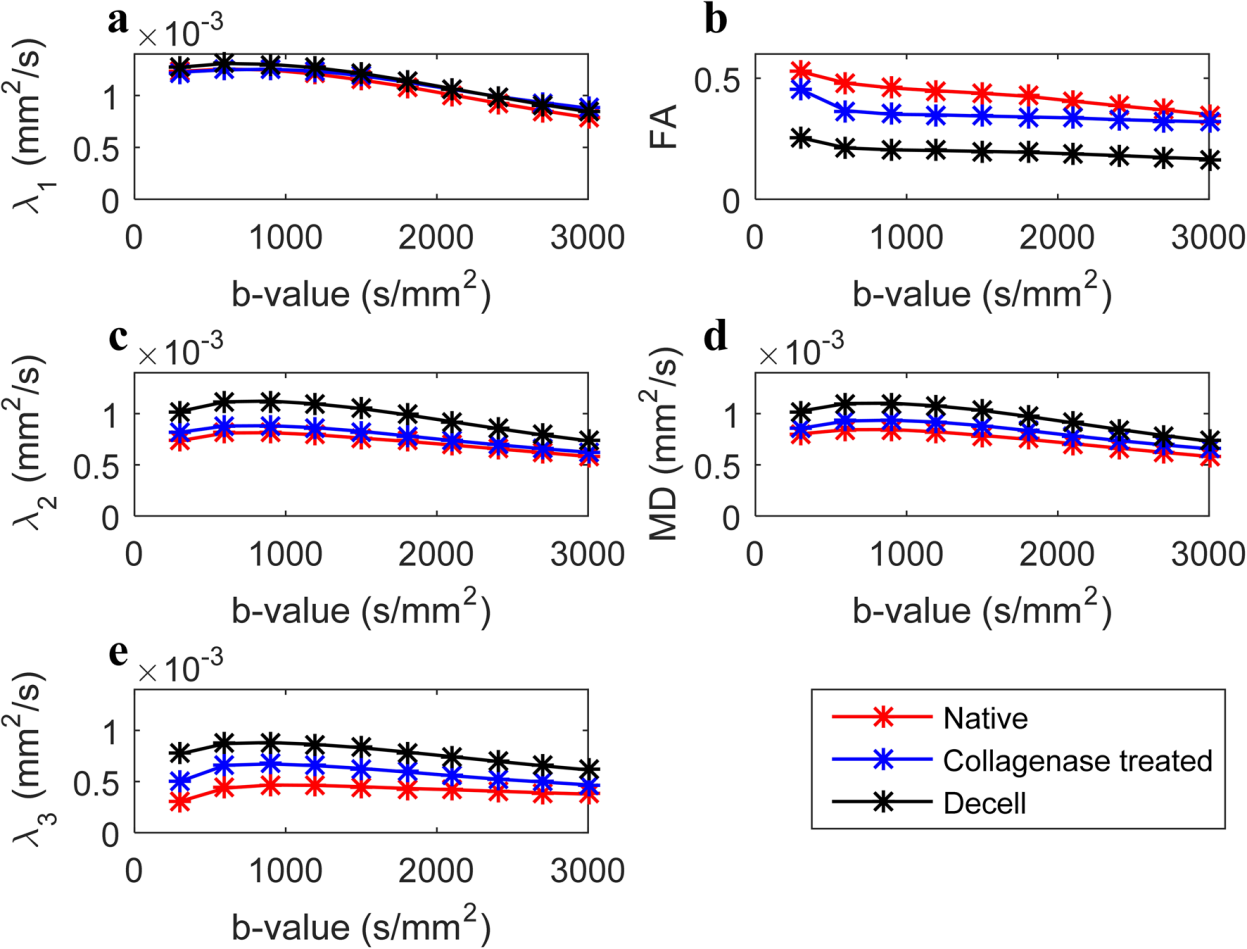
Effect of change in b-value by gradient strength on calculated eigenvalues (**a**, **c**, **e**), fractional anisotropy (**b**) and mean diffusivity (**d**). The analysis was carried out using HMb protocol.

Using HMb protocol, the influence of b-value (gradient strength) on the diffusion weighted signal across the three groups is shown in column 1 of Fig. 2. In each case, the attenuated signal from 6 different diffusion encoding directions are shown. A high degree of divergence in the 6 signal attenuation curves is observed in native and collagenase treated tissue samples, which is indicative of the underlying anisotropy and complexity of the tissues. This divergence in decay curves is lowest for the decellularized tissue. Column 2 in Fig. 2 shows the comparison of various signal representation based fitting schemes using arbitrary selected diffusion sensitizing gradient directions. Using HMb protocol, a tensor model was fit to data with b ≤ 900 s/mm^2^. DTI based directional and directionally invariant indices show significant differences between the native and treated samples (Fig. 3). The mean FA was highest in the native group and lowest in the decellularized group. Intra-group comparison of diffusivity along the eigenvectors shows lowest diffusivity along the direction of the tertiary eigenvector (Fig. 3e), whereas the inter-group comparison shows the lowest diffusivity in the native group. Figure 4 shows the effect of gradient strength on the eigenvalues, FA and MD of the calculated diffusion tensor using HMb DWIs. Among groups, eigenvalues (λ_1_, λ_2_ and λ_3_) from the decellularized samples were the highest across the b-values (Fig. 4a, c, e). The underestimation of eigenvalues, MD and overestimation of FA due to noise at low b-values (≤ 600 s/mm^2^) is shown in Fig. 4. In the Taylor expansion series of the diffusion attenuated signal (Supplementary Eq. 4), the first term represents a well-known mono-exponential model, whereas by including the second term, it becomes the quadratic exponential kurtosis model (Supplementary Eq. 6). The effect of ignoring the higher terms in Taylor series, i.e., only using the mono-exponential model (see supplementary *dMRI model fit* Eqs. 4, 6 and 9) will underestimate the signal fit at higher b-values. This behavior is evident at b > 1500 s/mm^2^ (Fig. 4).

**Figure 5:**
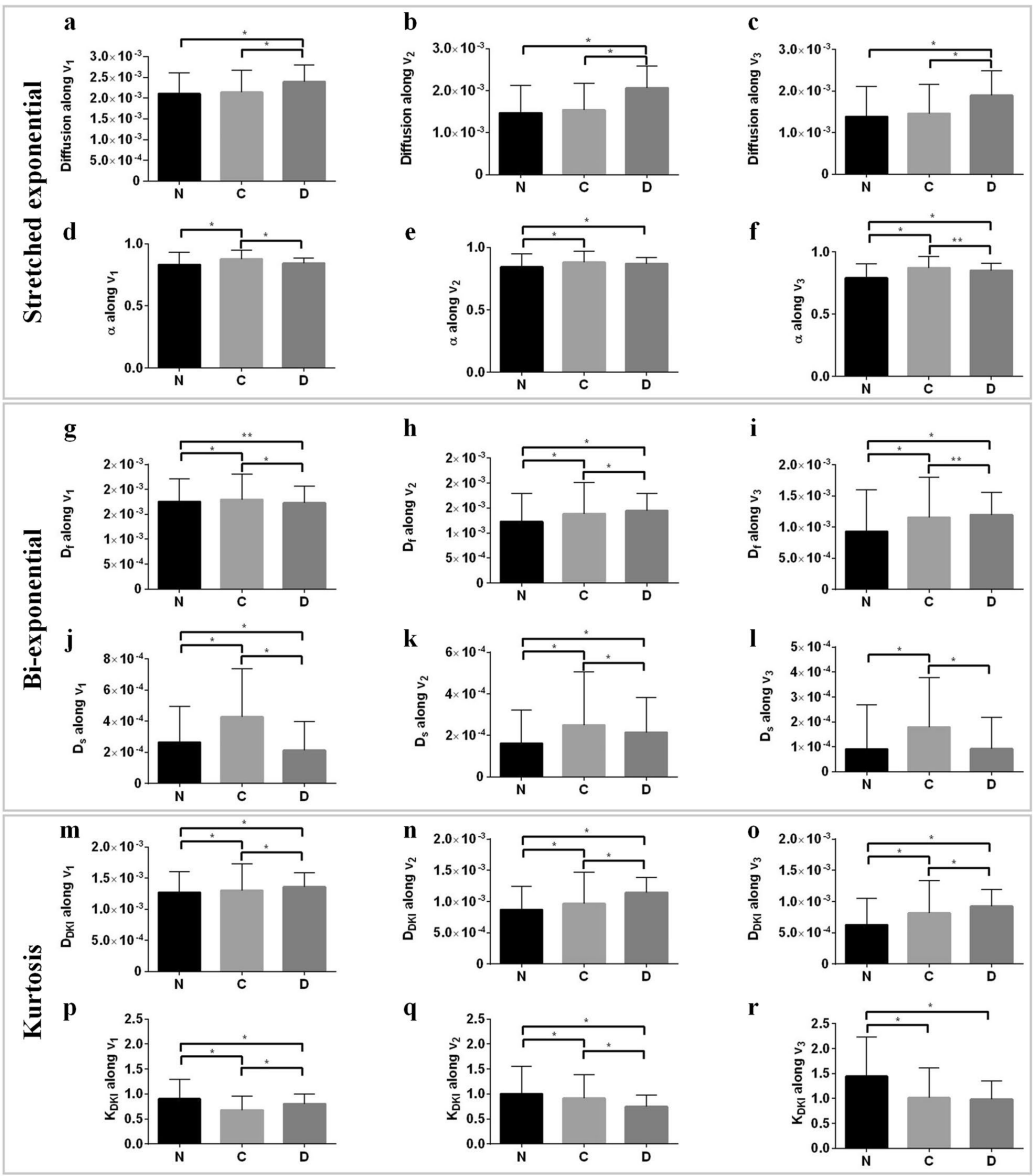
Quantification of tissue heterogeneity using non-Gaussian based parameters using HMb protocol. The derived indices are displayed as columns (mean) for each ROI. Three native tissue (N) samples, three collagenases treated tissue (C) samples and three decellularized tissue (D) samples were used for comparison. (n = 12, one ROI per slice, 4 slices per sample). (**a**–**f**) Stretched exponential, (**g**–**l**) Bi-exponential and (**m**-**r**) Kurtosis. Values represent mean ± SD. *p < 0.01; **p < 0.05.

To estimate the non-gaussian profile of native and treated tissues, stretched-exponential (b ≤ 3000 s/mm^2^), bi-exponential (b ≤ 3000 s/mm^2^) and diffusion kurtosis models (b ≤ 2400 s/mm^2^) were fit to the DWIs obtained using HMb protocol. Figure 5 shows the response from these three non-gaussian models. The stretched exponential model showed that, across the three tissue types, diffusivity is highest in the decellularized group and is significantly different from the native and collagenase treated samples (Fig. 5a–c). The heterogeneity index (α) was lowest in the native group with the highest degree of heterogeneity observed along the direction of the tertiary eigenvector (Fig. 5e–f). In the bi-exponential model, the fast diffusion components (Fig. 5g–i) exhibited similar trends as that observed from DTI based assessment (Fig. 3c–e). Quantitatively, the fast diffusivity profile across the groups indicates a statistically significant difference. Figure 5j–l show the diffusivity profile of slow diffusion components (D_s_) along the principal eigenvector (*v*_*1*_), secondary eigenvector (*v*_*2*_) and tertiary eigenvector (*v*_*3*_). Group-wise comparison showed that D_s_ is lowest in native tissue along v_2_ and v_3_, whereas collagenase treated tissues exhibited the highest diffusivity along *v*_*1*_–*v*_*3*_. Again group-wise comparison showed significant differences in D_s_ components among native and treated samples.

**Figure 6:**
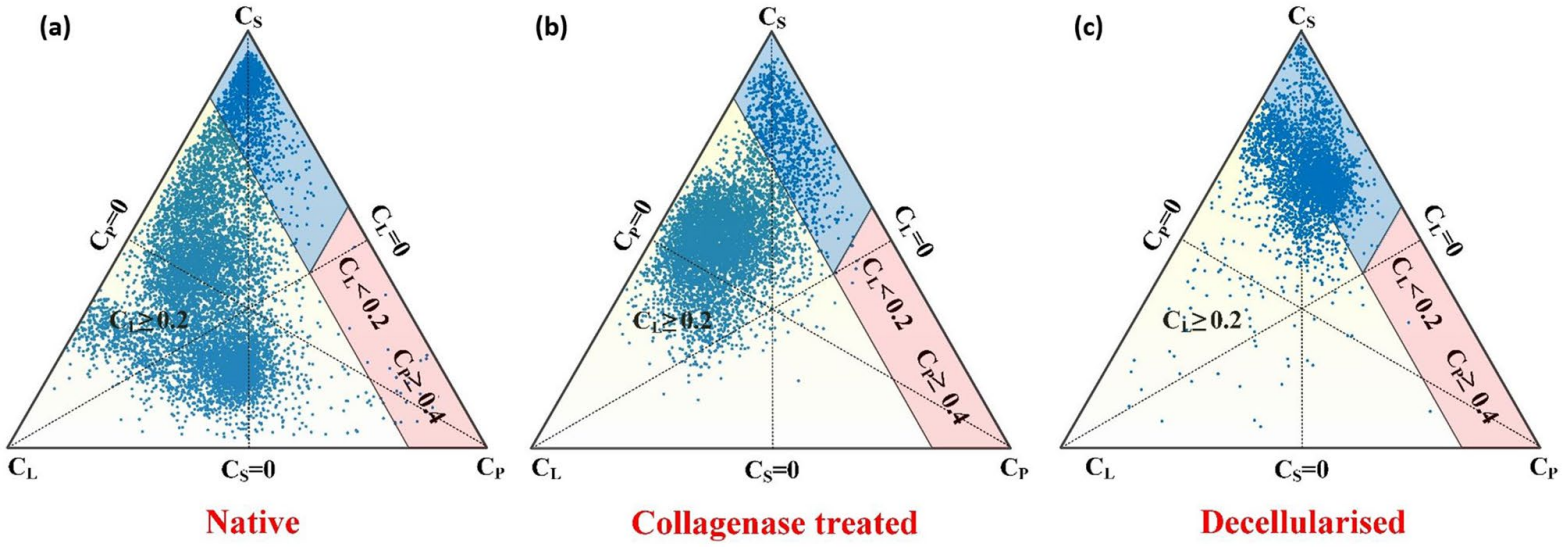
Comparative analysis of DTI based tensor shapes among (**a**) native, (**b**) collagenase treated and (**c**) decellularized tissue samples using HAR protocol. The diffusion/tensor profile is represented using barycentric space (3P). For this assessment, C_L,_ C_P_ and C_S_ were estimated from high angular-resolution DWI (b ≤ 800 s/mm^2^).

**Figure 7:**
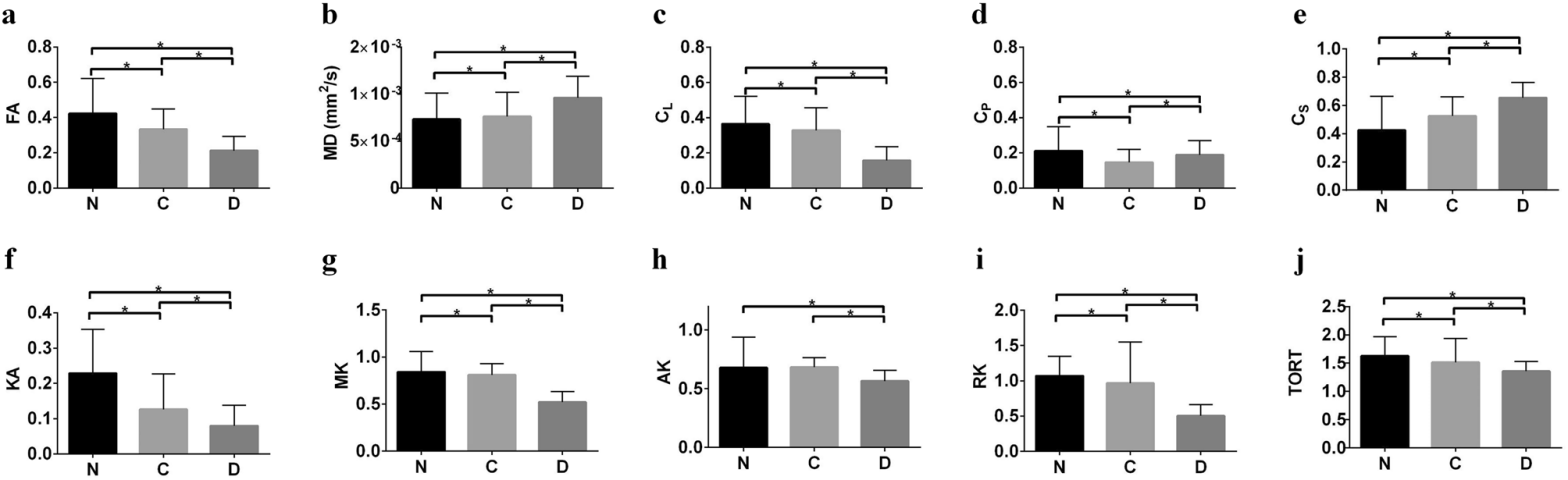
Quantification of changes in arterial tissue microstructural organization using HAR protocol. (**a**–**e**), DTI (b ≤ 800 s/mm^2^) based directionally invariant indices are displayed as columns (mean) for each ROI. (**f**–**j**), DKI (b ≤ 2500 s/mm^2^) based parameters are displayed as columns (mean) for each ROI. *KA* kurtosis anisotropy, *MK* mean kurtosis, *AK* Axial kurtosis, *RK* radial kurtosis, *TORT* tortuosity. Three native tissue (N) samples, three collagenase treated tissue (C) samples and three decellularized tissue (D) samples were used for comparison. (n = 12, one ROI per slice, 4 slices per sample). Values represent mean ± SD. *p < 0.05.

Figures 6 and 7a–e show the directionally invariant indices derived from the DTI model using HAR protocol based DWIs (b ≤ 800 s/mm^2^). For native tissue, the geometric measures in the 3P plot show a highly disperse shape distribution profile. The expanse of the shape distribution profile is indicative of the highly disperse (heterogeneous) nature of the underlying microstructural organization in native tissue (Fig. 6a). Compared to native, the shape distribution profile of collagenase treated group shows the absence of a cluster close to the C_L_–C_P_ line (Fig. 6b). In the decellularized group, the cluster is primarily confined close to C_S_ (Fig. 6c). Quantitatively, geometric measures, FA and MD all exhibited significant differences among the groups (Fig. 7a–e). DKI derived indices based on HAR protocol also exhibited significant differences between native and treated groups (Fig. 7f–j).

## Discussion and conclusion

In this study we explored the microstructural organization of arterial tissue using high spatial and angular resolution dMRI. To quantify the changes associated with the loss of collagen and SMC, diffusion profiles of native tissue, collagenase treated and decellularized tissue samples were mapped using gaussian and non-gaussian schemes. Gaussian and non-gaussian derived parameters were compared to estimate the sensitivity of dMRI to collagen and SMC.

In an arterial tissue the deterioration and depletion in extra-cellular matrix and or SMC may increase the local microstructural diffusivity. The alteration in diffusivity can be estimated by the mean squared displacement of water molecules at a given time. Using monoexponential signal representation, this change in diffusivity can be interpreted in terms of alterations in calculated directional diffusivity (eigenvalues) (Fig. 4a, c, e). In an unrestricted environment, this increase would be linear, however, in a hindered environment, the probability of interaction with cellular and extra-cellular media increases, resulting in reduced apparent diffusivity. Since vascular SMC are long and narrow structures which are enveloped by collagen, interaction of water molecules in such an environment tends to preferentially decrease the secondary and tertiary eigenvalues. As observed from Fig. 3, loss of collagen and SMC resulted in significant reduction in FA and a substantial increase in MD in the media of arterial tissue. Additionally, the diffusivity along the secondary and tertiary eigenvectors were significantly increased due to the removal of collagen and SMC. These results indicate that DTI (Gaussian) derived parameters are highly sensitive to changes in the microstructural composition of arterial tissue. Furthermore, DTI derived metrics such as those in the 3P plots (Fig. 6), and in particular C_L_ (Fig. 7), show the sensitivity as well as specificity of Westin indices to individual constituents of the arterial tissue.

Parameters derived from non-gaussian models also showed varying degrees of sensitivity to alterations in the microstructural composition of the arterial tissue. The diffusion profile derived from the stretched-exponential model showed significantly higher diffusivity in decellularized tissue, compared to the native and collagenase treated tissues. However, the model was not sensitive enough to distinguish changes associated with the loss of collagen. Hence, we can speculate that the stretched-exponential model is more specific to changes in the microstructure associated with SMC. The bi-exponential model was used to characterize microstructural changes in the arterial tissue due to two non-exchanging compartments with each compartment exhibiting a gaussian diffusivity profile. The results from the fast diffusion compartment can easily be related back to the results derived from the DTI model. The parameters derived from D_s_ showed that collagenase treated samples exhibited the highest degree of change in diffusivity along the primary, secondary and tertiary eigenvectors. On average the collagenase treated tissue showed approx. 1.5 times higher diffusivity, compared to the native samples. Since collagen in media forms a mesh like structure, enveloping SMCs^16^, it can be speculated that D_s_ derived parameters are more sensitive to microstructural changes associated with collagen than SMCs.

Greater complexity in microstructural organization of native tissue has also been shown by parameters derived from DKI (Figs. 5, 7). Lower diffusivity in the secondary and tertiary diffusion directions is indicative of greater restriction to molecular diffusion perpendicular to the cellular alignment. Kurtosis was highest in the tertiary diffusion direction due to increased heterogeneity in the radial direction. In contrast, kurtosis along the principal diffusion direction was lowest due to the circumferential alignment of SMCs, leading to a less restricted environment^11^. In inter-tissue comparison, the kurtosis along v_1_ (Kv_1_) was lowest in collagenase treated tissue, whereas kurtosis along v_2_ (Kv_2_) and kurtosis along v_3_ (Kv_3_) were lowest in decellularized tissues. Hence, it is plausible that Kv_1_ is more sensitive to collagen organization, whereas Kv_3_ is more sensitive to SMC organization (Fig. 5).

Using the bi-exponential model, the results of this study suggest that for the effective diffusion time (τ) of 7.33 ms, the average slow and fast diffusion rate in native tissue is approximately, 1.3 × 10^–3^ mm^2^/s and 0.17 × 10^–3^ mm^2^/s, respectively, Fig. 6. Sensitivity to the scale of diffusion depends on the time interval in which the diffusion is being probed^53^. Under the assumption that local microstructural environment can be characterized by two non-exchanging compartments, using the 3D-root-mean square equation (r^2^ = 6Dτ)^54^, we can speculate that the scale of fast (*r*_*f*_) and slow (*r*_*s*_) diffusion is sensitive in the range of approximately 8 µm and 3 µm, respectively. Given that the cellular diameters (on average) in the media of carotid arteries are in the range of 10–20 µm^55^ and diameters of collagen fibers are approximately 3–6 µm^56^, we can speculate that D_s_ associated parameters would be more sensitive to changes in smaller structures such as collagen. Several studies have looked at the contribution to the diffusion weighted signal from multiple components in different tissues^1,5,6,47^. Generally, changes in the ADC values are considered to indicate the presence of different structures or microstructural compartments^1,2,53^. However, to characterize the component specific contribution to the signal from different scales, higher order fitting and most commonly bi-exponential fitting equation is used^47,57^. Scale of diffusion from multiple compartments can then be associated to local microstructural environment based on their calculated fast and slow diffusivity coefficients. In particular, the fast diffusion contribution is often attributed to the extracellular space^57^, however, this behavior does not fully characterize the diffusion in biological tissues as water can exhibit non-gaussian diffusion which is not accounted for in mono- and bi-exponential models^1,46,53,58^. DKI, on the other hand, provides further characterization of the microstructure by incorporating non-gaussian diffusion in the diffusion model^46^. 

Diseases such as hypertension, atherosclerosis, aneurysms and aortic dissection cause a major change in the microstructural organization of arterial tissue. The effect of such changes on the mechanics of tissue have been demonstrated by several biomechanical studies^33^ (review^15^ and references therein). Such changes may alter the composition of collagen, elastin, GAG and SMC. A recent study by Tornifoglio et al.^31^ demonstrated the sensitivity of DTI to microstructural constituents of arterial tissue by selectively removing SMCs, collagen and elastin. Using mono-exponential (gaussian) approximation, the study showed SMCs to be the main contributor in attenuating the diffusion signal. Previous ex-vivo and in-vivo imaging studies have also demonstrated the efficacy of dMRI in characterizing atherosclerotic plaque fibrous caps, lipid-rich necrotic core and normal tissue^25–30^. In atherosclerosis, for example, the content and distribution of lipids play an important role in plaque formation. dMRI has been shown to improve the contrast between lipids and fibrous tissue^28^. Even though, this study did not analyze atherosclerotic plaques, nevertheless, the proposed methodology can be applied directly to such cases.

As demonstrated in this study, our MD based results from native tissue were comparable to the results of these above-mentioned imaging studies. However, most of these studies did not employ either high-angular resolution or high gradient strength (b-values) to map the restricted diffusivity profile of healthy or diseased tissues. To the best of our knowledge, this is the first study to-date that has employed higher-angular resolution and higher b-values to quantify the non-gaussian (restricted) behavior of arterial tissue microstructural organization and change in the composition due to the loss of collagen and SMC.

In this study three samples from three treatments were included. The low number of samples may limit statistical power, however the number was sufficient to identify systematic differences between each treatment type using both the gaussian and non-gaussian signal representation schemes. The results of this study were also complemented with histology. Multiple staining techniques were used to ensure the selective digestion of required constituents without compromising the internal structure of the tissue. Whilst the results from this study clearly demonstrate the critical role that cells play in the diffusion profile of arterial tissue, they also help to explain previous studies where different fixation techniques showed highly variable responses in dMRI. Studies by Flamini et al.^24^ and Tornifoglio et al.^31^ on arterial tissue and Agger et al.^59^ on myocardium, show how frozen tissue and formalin fixed tissue, respectively, induce significant alterations in the anisotropy of the tissue as compared to fresh tissue. This may be explained by cell necrosis due to ice crystal formation which is known to occur in frozen tissue^24,59^. In contrast, formalin fixed tissue may maintain tissue anisotropy by conserving cell volume and enabling extracellular diffusion^31^. This also highlights why the solution that the tissue is stored in would play a significant role in the diffusion response because it maintains the viability of the cells.

Whilst collagen is known to be important for the tensile strength of tissues^15^, it has also been identified that disrupted aortic plaque caps contain fewer SMCs. Given that SMCs are the collagen-synthesizing cell in tissue and plaques, fewer cells are most likely responsible for the reduced collagen content observed in compromised caps as compared to intact caps^60,61^. From this study, it is clear that reduced cell content could be identified in arterial tissue by the increased diffusivity in dMRI and may therefore offer a means to identify potentially vulnerable plaques in future. It is planned to explore this further in future work using dMRI of harvested human carotid endarterectomy plaques. It is worth noting that reduced anisotropy has been highlighted as a potential indicator of arterial disease however, to-date the means of exploring this was limited to ex vivo destructive techniques^62,63^. This study has demonstrated the sensitivity of higher-order diffusion schemes to microstructural alterations associated with the depletion of collagen and cell contents in arterial tissue and may present significant future biomarkers for the early identification of cardiovascular disease.

## Supplementary Information

Supplementary Information.

## Abbreviations

ADC: Apparent diffusion coefficient
AK: Axial kurtosis
CVD: Cardiovascular disease
dMRI: Diffusion magnetic resonance imaging
DKI: Diffusion kurtosis imaging
DTI: Diffusion tensor imaging
DWI: Diffusion weighted image
EPI: Echo planar imaging
FA: Fractional anisotropy
GAGs: Glycosaminoglycans
HAR: High angular resolution
HMb: High multi b-value
H&E: Hematoxylin and eosin
KA: Kurtosis anisotropy
MD: Mean diffusivity
MK: Mean kurtosis
PCaAs: Porcine carotid arteries
PBS: Phosphate buffer solution
PSR: Picro Sirius red
RK: Radial kurtosis
ROI: Region of interest
SHG: Second harmonic generation
SMC: Smooth muscle cell
SNR: Signal to noise ratio
TE: Echo time
TR: Repetition time
