# Supplementary material for: Exploring arterial tissue microstructural organization using non-Gaussian diffusion magnetic resonance schemes
Source: Sci Rep. 2021 Nov 15;11:22247. doi: 10.1038/s41598-021-01476-z (PMC8593063; doi:10.1038/s41598-021-01476-z)
Supplement: Supplementary file 1 — Supplementary Information. [file 41598_2021_1476_MOESM1_ESM.docx]

**SUPPLEMENTARY MATERIAL**

**This section has been adapted from Shahid, et al. [1].**

***dMRI modelling***

Under the assumption of infinitesimally short diffusion encoding gradient with amplitude G and duration δ, the signal attenuation in a pulsed gradient spin-echo (PGSE) sequence [2] is given by:

$$\frac{S(q,\Delta)}{S(0)}=\int P(R,\Delta)e^{2\pi iqR}dR$$

Where *q=γGδ/2π* is a wave vector [3], γ is the gyromagnetic ratio, *R* is the displacement the spins have experienced during the allowed diffusion time Δ, and *P(R)* is the ensemble average propagator (EAP). In 3D, it is a 3-dimensional probability density of diffusion (PDF) displacement. Regardless of the heterogeneity of the structure, the EAP provides averaged estimate of the diffusion environment. The diffusion profile can be obtained using the inverse Fourier transform of the signal with respect to the wave vector [3] and the excess kurtosis can be calculated using the following relation [4]:

$$K=\frac{\mu_{4}}{\mu_{2}^{2}}-3=\frac{\kappa_{4}}{\kappa_{2}^{2}}$$

Where *µ_i_*$=\int R^{n}P\left( R \right)dR,$the cumulants (*κ_i_*) can be described in terms of moments of probability distribution (*µ_i_*). The first three cumulants ( *κ_1-3_)* are equal to the first three moments ( *µ_1_*_-3_). *µ_1_, µ_2_* and *µ_3_* are the mean, variance and the skewness of the distribution, respectively. The fourth cumulant is related to the kurtosis as shown in eq. 2.

The diffusion Kurtosis can be derived from dMRI by using the Fourier relationship between the attenuated signal and the EAP. The logarithm of the diffusion signal can be expanded as a summation of the cumulants *κ_n_* of *P(R)[4]*:

$$\ln\frac{S(q)}{S(0)}=\sum_{p=1}^{\infty} \kappa_{n}\frac{\left(2\pi iqR\right)^{p}}{p!}$$

Under the assumption that diffusion is symmetrical (symmetric EAP), the phase of the attenuated signal can be considered zero, therefore, all odd order cumulants are null, i.e.,

$$\ln\frac{S(q)}{S(0)}=-\kappa_{2}\frac{\left(2\pi q\right)^{2}}{2}+\kappa_{4}\frac{\left(2\pi q\right)^{4}}{4!}+\kappa_{6}\frac{\left(2\pi q\right)^{6}}{6!}+...$$

For isotropic Gaussian diffusion in time Δ, the diffusion coefficient can be expressed as: *D=κ_2_/*(2Δ*)* [5], and by substituting it in eq. 2, the fourth cumulant can be written as: *κ_4_=4KD*^2^Δ^2^ .

Under the assumption of PGSE, the diffusion weighting parameter ‘b-value’ is defined as:

$$b=\gamma^{2}G^{2}\delta^{2}\Delta$$

Using the relations of the second and fourth cumulants and eq.5, the signal attenuation can be approximated by the quadratic exponential kurtosis model after truncating eq. 4 to the second term[5] :

$$\ln\frac{S(b)}{S(0)}\approx-bD_{app}+\frac{1}{6}b^{2}D_{app}^{2}K_{app}$$

Where, *b≈Δ(2πq)^2^*, and when *δ ≈ Δ* (violation of narrow pulse approximation), the effective diffusion time ‘τ’ ( τ = *Δ - δ/3*) should be used in Eq. 5 instead of *Δ*, i.e.,

$$b=\gamma^{2}G^{2}\delta^{2}\tau$$

By measuring the attenuated signal with multiple b-values, it is possible to estimate the apparent diffusivity (*D_app_*) and apparent diffusion kurtosis (*K_app_*) along a specific diffusion direction by fitting eq.6 (Supplementary Figures 1 and 2) [6]. The PDF of the diffusion kurtosis model (eq. 6) is a Gaussian distribution with mean (*D_app_*) and variance ($\frac{1}{3}D_{app}^{2}K_{app})$. Therefore, kurtosis can be used to estimate the degree of heterogeneity of the underlying microstructural environment.

The diffusion weighted signal vs. the b-value curve is a quadratic on a semi-log scale with minimum at $b=3/D_{app}K_{app}$ (Supplementary Figure 1). Therefore, for the DKI model to provide meaningful estimation on *D_app_* and *K_app_*, it is assumed that the diffusion weighted signal is a monotonically decreasing function of the b-value and this model is only valid for $b<3/D_{app}K_{app}$ [5].

In order to understand the influence of higher b-values on the diffusion weighted signal, a bi-exponential model was used. If the signal attenuation is attributed to two non-exchanging compartments with each compartment exhibiting Gaussian diffusion in such a way that one of the compartments exhibits fast diffusion (*D_f_*) and the second compartment exhibits slow diffusion (*D_s_*), then the bi-exponential model can be described as [7]:

$$\frac{S(b)}{S(0)}=v_{f}e^{-D_{f}b}+v_{s}e^{-D_{s}b}$$

Where *v_f_* and *v_s_* are the volume fraction corresponding to the fast and slow diffusion compartments, respectively. Under the assumption that *D_f_ ≥ D_s_*, the mean diffusivity (*D_biexp_*) and kurtosis (*K_biexp_*) can be calculated as: *D_biexp_= v_f_D_f_ + v_s_D_s_* and $K_{biexp}=3v_{f}v_{s}\left( D_{f}-D_{s} \right)^{2}/D_{biexp}^{2}$ [5].

For b-values that are significantly small (depending upon the type of tissue) and if a voxel contains a single compartment exhibiting homogeneous T2 relaxation, then for unrestricted Gaussian diffusion, the higher terms of eq. 4 can be neglected and eq.4 takes the form of a well-known mono-exponential (DTI) model [8]:

$$\ln\frac{S(b)}{S(0)}\approx-bD_{app}$$

By fitting eq.9 over a range of b-values, *D_app_* can be estimated. However, if the range of b-values is very small (maximum b-value is too low) then the attenuation in the signal intensity will be very low and the estimation of *D_app_* will be prone to noise. On the other hand, if the range is very large (max b-value is very high), then the estimation of diffusivity will incur a systematic error due to the omission of higher terms from eq. 4. Recently, dMRI studies on ex-vivo carotid artery have shown that, under the assumption of a single compartment with homogeneous T2 relaxation, the optimal b-value in close to 800 s/mm^2^ [9, 10].

In this study, a stretched exponential model has also been employed to examine the heterogeneity in the microstructural environment of arterial tissue. The model describes the signal attenuation as a continuous distribution from multiple compartments (sources) with variable rates of decay [11]. The model is defined as:

$$\frac{S(b)}{S(0)}=e^{-b^{\alpha}D_{st}}$$

Where α is a dimensionless stretching parameter or a heterogeneity index (0 < α ≤ 1) and *D_st_* is the stretched-regulated diffusivity. α characterizes the deviation of the diffusion-weighted signal from mono-exponential behavior. α close to 0 describes non-monoexponential behavior due to underlying microstructural complexity (heterogeneity), or in other words a higher degree of kurtosis, and α close to 1 indicates a higher degree of homogeneity in the diffusion profile.

**References**:

[1] S. S. Shahid, C. M. Kerskens, M. Burrows, and A. G. Witney, "Elucidating the complex organization of neural micro-domains in the locust Schistocerca gregaria using dMRI," *Scientific reports,* vol. 11, no. 1, pp. 1-12, 2021.

[2] E. O. Stejskal and J. E. Tanner, "Spin diffusion measurements: spin echoes in the presence of a time‐dependent field gradient," *The journal of chemical physics,* vol. 42, no. 1, pp. 288-292, 1965.

[3] J. Latt *et al.*, "In vivo visualization of displacement-distribution-derived parameters in q-space imaging," *Magnetic resonance imaging,* vol. 26, no. 1, pp. 77-87, Jan 2008, doi: 10.1016/j.mri.2007.04.001.

[4] V. G. Kiselev, "The Cumulant Expansion: An Overarching Mathematical Framework For Understanding Diffusion NMR," in *Diffusion MRI: Theory, Methods and Applications*, D. K. Jones Ed., (Diffusion MRI: Theory, Methods and Applications, D. K. Jones, Ed.: Oxford University Press, 2010.

[5] J. H. Jensen and J. A. Helpern, "MRI quantification of non-Gaussian water diffusion by kurtosis analysis," *NMR Biomed,* vol. 23, no. 7, pp. 698-710, Aug 2010, doi: 10.1002/nbm.1518.

[6] J. H. Jensen, J. A. Helpern, A. Ramani, H. Lu, and K. Kaczynski, "Diffusional kurtosis imaging: the quantification of non-gaussian water diffusion by means of magnetic resonance imaging," *Magn Reson Med,* vol. 53, no. 6, pp. 1432-40, Jun 2005, doi: 10.1002/mrm.20508.

[7] C. A. Clark and D. Le Bihan, "Water diffusion compartmentation and anisotropy at high b values in the human brain," *Magnetic Resonance in Medicine: An Official Journal of the International Society for Magnetic Resonance in Medicine,* vol. 44, no. 6, pp. 852-859, 2000.

[8] E. Stejskal, "Use of spin echoes in a pulsed magnetic‐field gradient to study anisotropic, restricted diffusion and flow," *The Journal of Chemical Physics,* vol. 43, no. 10, pp. 3597-3603, 1965.

[9] S. Salman Shahid, R. T. Gaul, C. Kerskens, V. Flamini, and C. Lally, "Quantifying the ultrastructure of carotid arteries using high-resolution micro-diffusion tensor imaging---comparison of intact versus open cut tissue," *Physics in Medicine and Biology,* vol. 62, p. 8850, 2017.

[10] V. Flamini, C. Kerskens, K. M. Moerman, C. K. Simms, and C. Lally, "Imaging Arterial Fibres Using Diffusion Tensor Imaging—Feasibility Study and Preliminary Results," *EURASIP Journal on Advances in Signal Processing,* vol. 2010, no. 1, pp. 1-13, 2010.

[11] K. M. Bennett, K. M. Schmainda, R. T. Bennett, D. B. Rowe, H. Lu, and J. S. Hyde, "Characterization of continuously distributed cortical water diffusion rates with a stretched-exponential model," *Magn Reson Med,* vol. 50, no. 4, pp. 727-34, Oct 2003, doi: 10.1002/mrm.10581.

**SUPPLEMENTARY FIGURES**


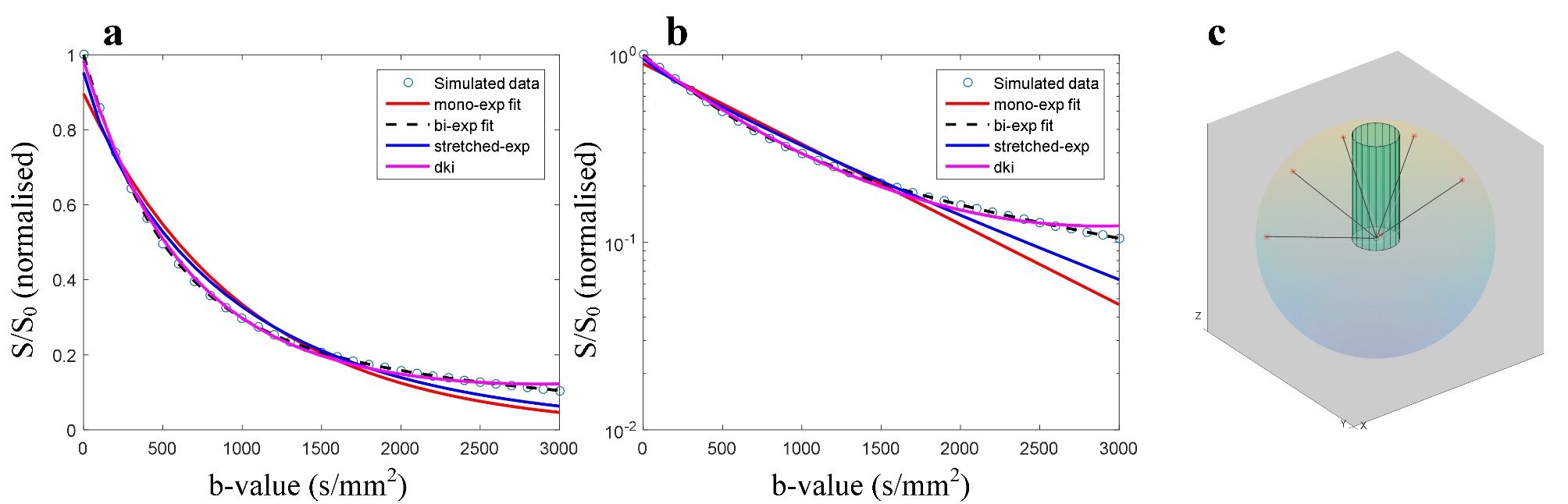


**Supplementary Figure 1:** Comparison of various Signal representation based fitting schemes on simulated data. The data was generated using a bi-exponential model with D*_f_* = 1.4 x10^-3^ mm^2^/s, D*_s_* = 0.50x10^-3^ mm^2^/s, *v_f_* = 0.7 and *v_s_* = 0.3. (a) Normalized signal intensity versus the b-values. The range of b-values is from 0 to 3000 s/mm^2^. Mono-exponential and stretched-exponential fits tend to deviate from the simulated signal decay curve for b ≥ 1000 s/mm^2^. (b) Semi-log representation of signal decay curve as a function of b-values. The signal representation-based fits are also represented. The deviation of various fits from the simulated signal decay curve is much more prominent for b-values ≥ 1500 s/mm^2^. (c) An illustration of the six-diffusion sensitizing gradient directions (only the positive hemisphere) used for multi b-value diffusion-weighted images.


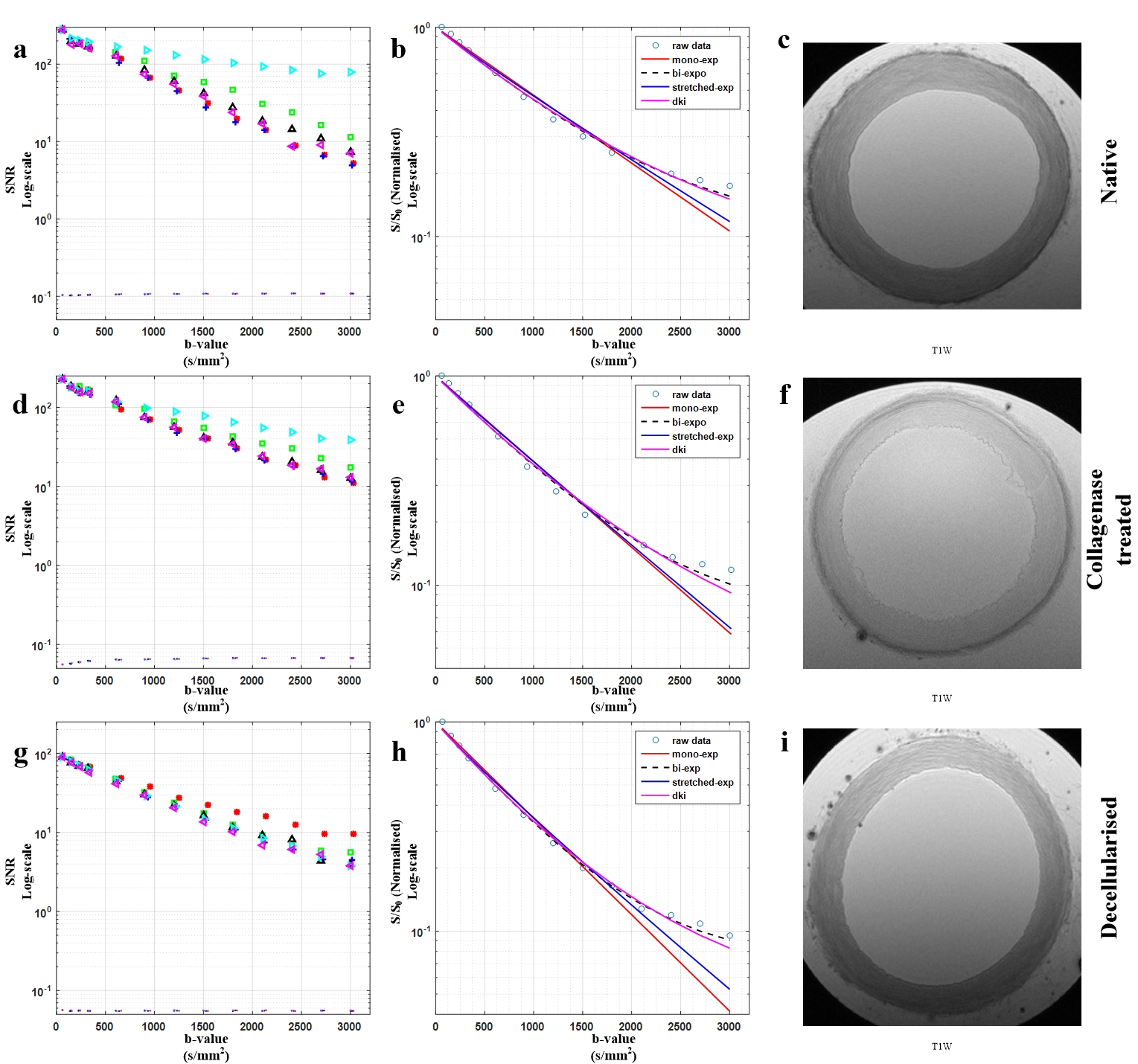


**Supplementary Figure 2:** SNR of denoised Diffusion-weighted signal decay curves as a function of b-value from six non-collinear diffusion-encoding directions (HMb protocol). SNR profile of diffusion-weighted signal decay curve across six diffusion-encoded directions for (a) native tissue, (d) collagenase treated tissue and (g) decellularized tissue sample. In a, d and g, mean background signal intensity is also shown. b, e and f, show the comparison of various signal representation based fitting schemes on the denoised diffusion-weighted signal (for illustration purpose, the DW-signals from the sixth diffusion-encoded direction were used in b, e and h). c, f and i show the high-resolution T1-weighted images of native, collagenase treated and decellularized tissues samples, respectively.

Supplementary Figure 3: Sample raw DWI data showing the effect of diffusion weighting on a single slice of native tissue sample (for illustration purpose, the raw DWI from the sixth diffusion-encoded direction were used).

Supplementary Figure 4: Sample denoised DWI data showing the effect of diffusion weighting on a single slice of native tissue sample (for illustration purpose, the denoised DWI from the sixth diffusion-encoded direction were used).
